# Integrating Psychosocial Screening Into Cardiac Nursing: Feasibility of Nurse‐Led Type D Personality Assessment for Predicting Emotional Recovery in Coronary Care Unit Patients

**DOI:** 10.1111/nicc.70268

**Published:** 2025-12-16

**Authors:** Anastasia Lykou, Evangelos C. Fradelos, Ioanna Dimitriadou, Maria Saridi, Eustratia Mourtou, Pavlos Sarafis, Dimos Mastrogiannis, Ioanna V. Papathanasiou, Aikaterini Toska

**Affiliations:** ^1^ School of Social Sciences Open Hellenic University Patras Greece; ^2^ Department of Nursing University of Thessaly Larissa Greece; ^3^ Department of Information Systems Directorate of Informatics Patras Greece; ^4^ Department of Nursing University of West Attica Athens Greece

**Keywords:** anxiety, cardiac nursing, coping, coronary care, depression, emotional recovery, type D personality

## Abstract

**Background:**

Psychological factors such as anxiety, depression and Type D (distressed) personality have been associated with poor prognosis and slower emotional recovery in patients with coronary heart disease. Despite this evidence, systematic psychosocial screening remains limited in cardiac nursing practice.

**Aim:**

The aim of this study was to evaluate the feasibility of nurse‐led Type D personality (TDP) screening and its potential role in predicting emotional recovery among patients hospitalised in a coronary care unit (CCU).

**Study Design:**

A cross‐sectional observational study was conducted. Data were collected through validated self‐report questionnaires assessing TDP, coping strategies, anxiety and depression. Statistical analyses included descriptive statistics, correlation analyses and multiple linear regression.

**Results:**

110 CCU patients took part in the study. Type D personality was identified in (*n* = 32.5%) of participants. Patients with TDP demonstrated significantly higher levels of anxiety and depression and more frequent use of maladaptive coping strategies. Regression analyses revealed that TDP and avoidant coping were independent predictors of anxiety and depression scores, explaining a significant proportion of variance in emotional outcomes.

**Conclusions:**

Nurse‐led psychosocial screening for TDP is feasible in the CCU setting and provides valuable information for identifying patients at risk of poor emotional recovery. Integrating this assessment into nursing care may promote holistic management and improve psychological support during cardiac rehabilitation.

**Relevance to Clinical Practice:**

Routine psychosocial screening by nurses can facilitate early detection of distressed cardiac patients and guide targeted interventions. Incorporating Type D personality assessment into standard nursing procedures may enhance emotional recovery, reduce psychological morbidity and contribute to comprehensive cardiac care.

**Impact Statements**

- What is known about the topic
  ○ Type D personality and poor coping strategies are associated with anxiety, depression and poor outcomes in cardiac patients.
  ○ Routine psychological assessment in coronary care units (CCUs) is rarely performed despite recommendations from clinical guidelines.
  ○ Emotional distress can delay recovery and reduce engagement in cardiac rehabilitation.
- What this paper adds
  ○ Demonstrates the feasibility of nurse‐led Type D personality and coping assessment in a CCU setting.
  ○ Identifies Type D personality and avoidant coping as independent predictors of anxiety and depression.
  ○ Supports integrating psychosocial screening into nursing care for early detection of vulnerable cardiac patients.

## 1 Introduction

The prevalence of cardiovascular diseases (CVDs) is steadily increasing, currently affecting an estimated 46.4% of the global population [1]. Beyond the significant health implications for patients, CVDs also have far‐reaching consequences on personal and professional life, placing substantial financial strain on both individuals and healthcare systems [2, 3]. In addition to well‐established risk factors, psychological conditions such as anxiety, depression and Type D personality (TDP) have emerged as important contributors to both the onset of heart disease and poor cardiovascular outcomes in patients with coronary artery disease (CAD) [4, 5].

Despite the high prevalence of clinically significant symptoms of depression and anxiety among patients with CAD, psychological treatments have shown limited effectiveness in managing these symptoms. Current interventions often yield minimal therapeutic benefit, with little to no impact on cardiac prognosis [6, 7, 8]. Routine psychological assessment remains underutilised in coronary care units (CCUs), where the primary focus remains on physiological stabilisation rather than on psychosocial recovery. In recognition of this, the latest European clinical practice guidelines for the prevention of CVD recommend routine screening for depression, anxiety and TDP in patients with CAD. These psychological factors are strongly associated with unhealthy lifestyle behaviours, poor adherence to treatment and reduced engagement in cardiac rehabilitation programmes [9].

## 2 Background

Type D personality is characterised by a combination of high negative affectivity (NA)—the persistent tendency to experience negative emotions such as anxiety, stress, and tension—and high social inhibition (SI), which involves a reluctance to express oneself in social contexts due to fear of disapproval or negative evaluation [10]. Studies have reported a high prevalence of current and past major depressive episodes among individuals with TDP, as well as moderate‐to‐strong correlations between the NA component of TDP and anxiety symptoms [11, 12, 13].

Coping strategies have also been identified as potentially valuable tools in the management of psychological distress among patients with CVD. Defined by Lazarus et al. as a set of behavioural and cognitive efforts aimed at mitigating the effects of stressful life events [14], coping strategies can play a significant role in reducing anxiety and depression. Evidence suggests that coping skill training is associated with marked improvements in anxiety levels [15], emphasising the importance of incorporating these strategies into the management of patients with CAD [16].

Cardiovascular nurses are key players in the ongoing assessment and emotional support of hospitalised cardiac patients. However, few studies have investigated how personality profiling could be practically integrated into cardiac nursing practice to guide timely psychological intervention. In particular, the relationships between TDP, coping strategies and emotional recovery in patients with chronic heart failure remain unexplored in Mediterranean clinical settings.

## 3 Aims and Objectives

To the best of our knowledge, no prior studies have examined the prevalence or clinical significance of TDP and coping strategies among cardiac patients in Greece. This study aimed to (1) determine the prevalence of TDP among CCU patients, (2) examine its association with anxiety, depression and coping strategies and (3) explore the potential of nurse‐led personality profiling as a screening strategy for early psychosocial risk detection in cardiac care.

## 4 Design and Methods

### 4.1 Setting and Sample

This cross‐sectional study was conducted in the CCU of the General Hospital of Rhodos, between October and December 2023. The research followed the Strengthening the Reporting of Observational Studies in Epidemiology guidelines for cross‐sectional studies. The purpose of the study was to explore the relationship between TDP, coping strategies and emotional distress in patients hospitalised for acute cardiac events, with the broader goal of integrating personality profiling into nursing assessment and care.

The study population consisted of adult patients aged 18 years or older who were hospitalised in the CCU for acute coronary or ischaemic cardiac conditions. Eligible participants were those who were clinically stable, able to communicate in Greek and capable of completing self‐administered questionnaires. Exclusion criteria included severe psychiatric disorders, cognitive impairment or medical instability that could interfere with participation. A consecutive sampling strategy was employed to minimise selection bias and ensure that the sample represented the hospitalised cardiac population of the unit.

### 4.2 Data Collection Tools and Methods

Data were collected by a single trained cardiac nurse to ensure methodological consistency and minimise inter‐observer variation. The nurse approached eligible patients after medical stabilisation, explained the study's purpose, obtained written informed consent and assisted with the administration of the questionnaires. Data collection took place within 48 to 72 h of hospital admission to capture early psychological reactions while patients remained in the CCU. The nurse ensured a supportive and calm environment, clarifying any questions and assisting participants when needed. Each assessment required approximately 20 min to complete. The use of a single data collector helped maintain uniformity in administration procedures and enhanced data reliability.

Data were collected using a structured questionnaire that included sociodemographic information and three validated psychometric instruments: the Hospital Anxiety and Depression Scale (HADS), the Type D Personality Scale (DS14) and the Brief COPE Inventory. All tools had been previously validated in Greek clinical populations and were selected for their brevity, reliability and suitability for use in hospital settings.

#### 4.2.1 Hospital Anxiety and Depression Scale (HADS)

The HADS, developed by Zigmond and Snaith [17], was used to assess anxiety and depressive symptoms. It consists of 14 items divided equally between two subscales—anxiety and depression—each rated on a 4‐point Likert scale ranging from 0 to 3. Higher scores reflect greater levels of psychological distress. The Greek version, validated by Michopoulos et al. [18], has demonstrated strong psychometric properties, with Cronbach's alpha values ranging from 0.80 to 0.85 and test–retest reliability coefficients of *r* = 0.84. In this study, internal consistency was excellent, with Cronbach's α = 0.86 for the anxiety subscale and α = 0.82 for the depression subscale. The HADS is a practical, nurse‐administered tool that can be completed in less than 10 min and excludes somatic items that might overlap with cardiac symptoms.

#### 4.2.2 Type D Personality Scale (DS14)

The DS14, developed by Denollet [10], measures two stable personality dimensions—NA and SI—each consisting of seven items rated on a 5‐point Likert scale (0–4). A score of 10 or higher on both subscales indicates the presence of TDP. The Greek version, adapted by Kontodimopoulos et al. [19], has shown excellent psychometric validity and internal consistency (Cronbach's α = 0.88 for NA and 0.86 for SI). In this study, reliability remained high (α = 0.89 for NA and α = 0.84 for SI). The DS14 is a brief instrument, requiring only two to 3 min to complete and can be readily integrated into nursing admission assessments to identify patients with higher emotional vulnerability.

#### 4.2.3 Brief COPE Inventory

Coping strategies were evaluated using the Brief COPE Inventory (Carver) [20], a 28‐item questionnaire assessing adaptive and maladaptive coping behaviours. The instrument evaluates 14 coping responses that can be categorised into three higher order domains: problem‐focussed coping, emotion‐focussed coping and avoidant coping. Responses are rated on a 4‐point scale ranging from 1 (‘I haven't been doing this at all’) to 4 (‘I've been doing this a lot’). The Greek version validated by Kapsou et al. (2010) [21] has demonstrated adequate psychometric properties, with Cronbach's alpha coefficients ranging from 0.78 to 0.87. In this study, Cronbach's alpha values ranged from 0.76 to 0.88 across the three domains. The Brief COPE can be administered within 10 min and provides valuable insight into patients' stress management styles, which can inform targeted nursing interventions.

To ensure data integrity and reliability, all questionnaires were checked immediately after completion by the nurse‐researcher. Data were then double‐entered into a secure electronic database and cross‐verified by a second researcher to identify potential discrepancies. Missing data were minimal (below 3%) and were addressed using pairwise deletion. The use of standardised, validated instruments and a single trained data collector helped minimise measurement bias and enhance internal consistency.

### 4.3 Data Analysis

Data analysis was performed using IBM SPSS Statistics, version 26. Descriptive statistics were used to summarise participants' sociodemographic and psychological characteristics, with mean and standard deviation calculated for continuous variables and frequencies and percentages for categorical variables. The normality of continuous data was examined using the Kolmogorov–Smirnov test. Pearson's correlation coefficients were computed to examine associations among anxiety, depression, TDP and coping strategies. Multiple linear regression analyses were then performed to identify independent predictors of anxiety, depression and total HADS scores after adjusting for demographic covariates, including age, gender, educational level and prior history of coronary heart disease (CHD). Statistical significance was defined as *p* < 0.05, and all tests were two‐tailed. Internal consistency for each psychometric tool was confirmed by calculating Cronbach's alpha coefficients, which demonstrated satisfactory reliability across all scales. Multicollinearity diagnostics (Variance Inflation Factor; VIF) indicated acceptable levels for all predictor variables (VIF < 3.0), suggesting no serious collinearity issues.

### 4.4 Ethics and Institutional Approvals

Ethics approval was obtained from the Research Ethics Committee of the General Hospital of Rhodes (Protocol No. 28056/8‐10‐2023). All procedures were conducted in accordance with the ethical principles of the Declaration of Helsinki. Participants received both oral and written information about the study and provided written informed consent prior to participation. Anonymity and confidentiality were strictly maintained through coded identifiers, and participants were assured that their decision to participate or withdraw would not affect their medical treatment or nursing care.

## 5 Results

A total of 118 patients were screened during the study period, of whom 110 met the inclusion criteria and agreed to participate, yielding a response rate of 93.2%. A total of 110 patients participated in the study (*n* = 110). The gender distribution was nearly equal, with 49.1% male and 50.9% female participants. The majority were married (73.6%), whereas 14.5% were single. In terms of family structure, 40.9% had two children, whereas 20.9% reported having no children. Educational attainment was predominantly at the tertiary level (64.5%), followed by secondary (24.5%) and primary education (10.9%). Regarding employment status, 70.9% of participants were employed, and 25.5% had a history of CHD. These demographic characteristics are summarised in Table 1.

**TABLE 1:** Sociodemographic and clinical characteristics of CCU patients (*n* = 120).

Gender	Man	54	49,1
	Woman	56	50,9
Age (mean)	51.127		
Marital status	Unmarried	16	14,5
	Married	81	73,6
	Divorced	9	8,2
	Widower	4	3,6
Number of children	0	23	20,9
	1	28	25,5
	2	45	40,9
	3	11	10
	4	2	1,8
	5	1	0,9
Educational level	Primary education	12	10,9
	Secondary education	27	24,5
	Tertiary education	71	64,5
Laboral status	Working	78	70,9
	Unemployed	4	3,6
	Retired	28	25,5
History of Coronary Disease (CD)	Yes	16	14,5
	No	94	85,5

*Note:* Summary of participants' demographic data (age, gender, marital status, education and occupation) and clinical characteristics (diagnosis, hospitalisation reason, comorbidities and lifestyle factors).

**FIGURE 1:**
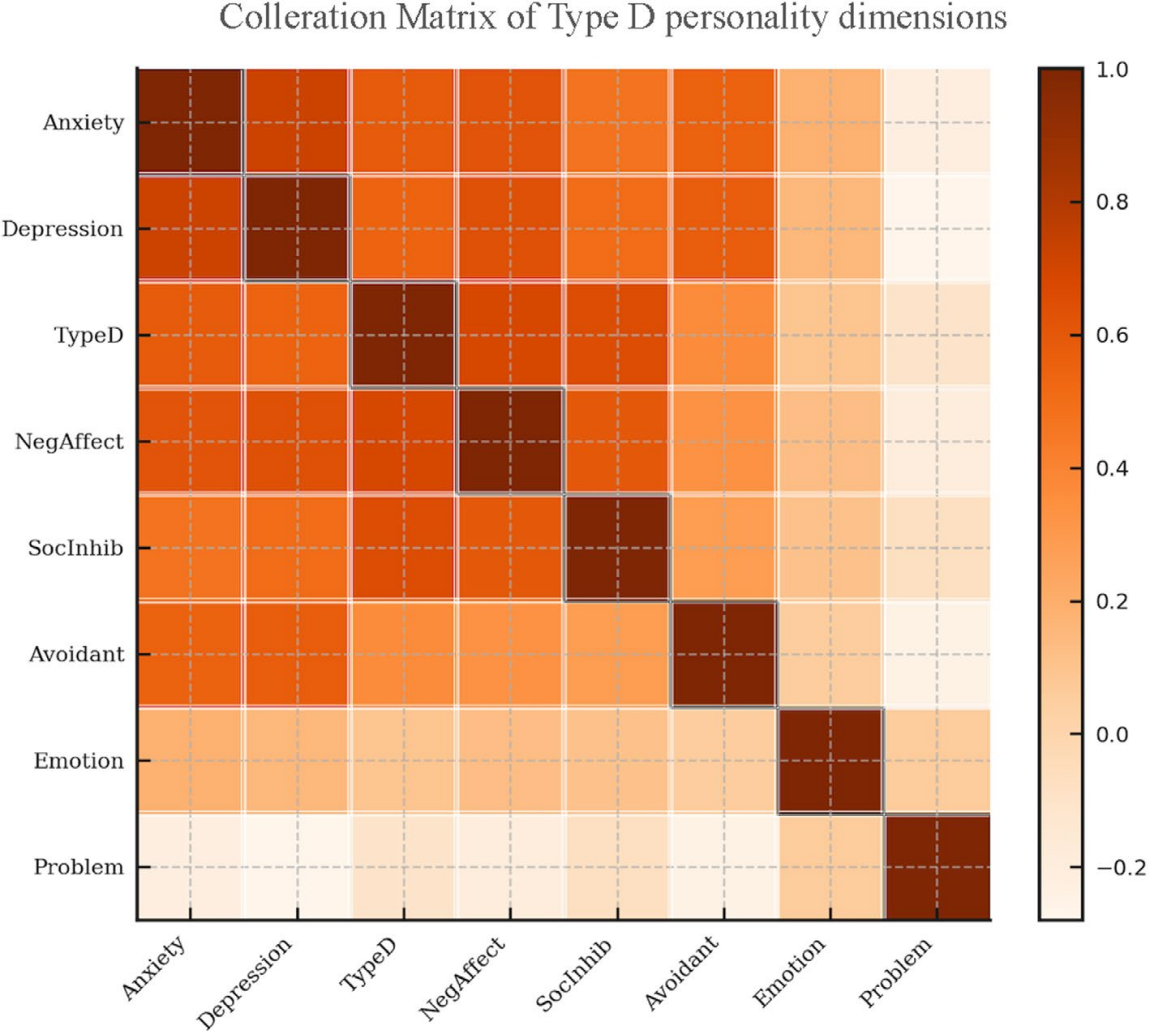
Correlation matrix of Type D personality dimensions, coping strategies, anxiety and depression in coronary care unit patients. Warmer shades indicate stronger positive correlations. Exact correlation coefficients are reported in Table 2. Cells along the diagonal highlight variable identity structure.

Table 2 presents the descriptive statistics of the scales used in the study. The mean score for anxiety was 8.01 (SD = 4.62), with values ranging from 0 to 18. Depression had a mean score of 7.65 (SD = 3.99) and a range from 0 to 17. The total score of the HADS was 15.66 (SD = 7.71), with values ranging from 0 to 32. The mean score for negative emotionality was 10.10 (SD = 6.30), whereas SI had a mean value of 10.32 (SD = 4.54). Personality type D presented a mean value of 20.42 (SD = 8.95), with values from 4 to 37. Regarding coping strategies, the mean value for problem‐focussed coping was 21.89 (SD = 3.54), for emotion‐focussed coping 30.02 (SD = 4.59) and avoidant coping 14.57 (SD = 3.91). The values for coping strategies had narrower ranges, indicating smaller variations among participants. As shown in Figure 1, variables related to emotional distress (anxiety and depression) demonstrate stronger correlations with NA and avoidant coping, whereas problem‐focussed coping shows weaker or negative associations.

**TABLE 2:** Descriptive statistics and correlations among type D personality, coping strategies, anxiety and depression.

	Mean	Std. deviation
Anxiety	8.009	4.620
Depression	7.645	3.999
Total_Hospital Anxiety Depression Scale	15.655	7.711
Negative Affectivity	10.100	6.298
Social Inhibition	10.318	4.543
Personlity Type_D	20.418	8.946
Problem_Focussed_Coping	21.891	3.538
Emotion_Focussed_Coping	30.018	4.588
Avoidant_Coping	14.573	3.906

*Note:* Mean scores and standard deviations for study variables, and Pearson's correlation coefficients showing associations between Type D personality, coping styles, anxiety and depression levels.

Regarding anxiety, 59% (*n* = 52) were found to be at normal levels, 22% (*n* = 27) had moderate levels, and 26.4% (*n* = 31) had high levels. Regarding depression, 53.6% (*n* = 59) were in the normal levels category, 20% (*n* = 22) had moderate levels, and 26.4% (*n* = 29) were classified as high levels.

**FIGURE 2:**
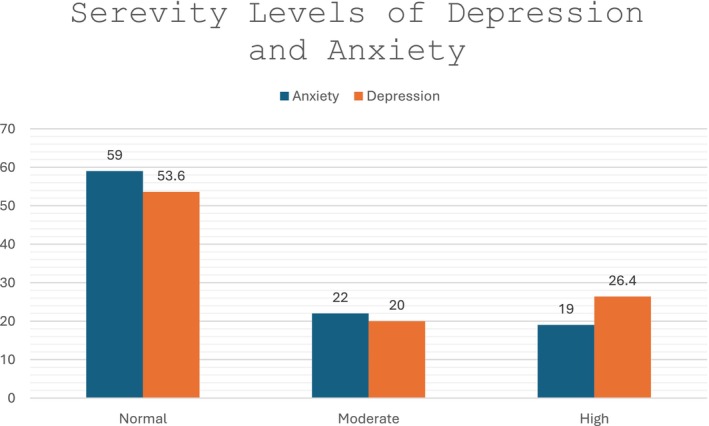
Bar plot illustrating the distribution of anxiety and depression severity levels among coronary care unit patients. Percentages are based on HADS severity thresholds.

Table 3 presents the results of the linear regression with the dependent variable being the HADS scores and the independent variables being Personality Type D and coping strategies, adjusted for demographic characteristics. Personality Type D was a significant predictor of anxiety (*b* = 0.362, *p* < 0.001), explaining 56.6% of the variance (*F* (10,109 109) = 12.971, *p* < 0.001). Coping strategies did not have a statistically significant effect. Personality Type D was positively associated with depression (*b* = 0.188, *p* < 0.001). Avoidant coping also had a positive association (*b* = 0.265, *p* < 0.001), whereas problem‐focussed coping had a negative effect (*b* = −0.218, *p* = 0.035). The model explained 62.9% of the variance (*F* (10,109 109) = 18.874, *p* < 0.001). Personality Type D had the strongest effect on the total score (*b* = 0.535, *p* < 0.001), whereas avoidant coping also contributed positively (*b* = 0.399, *p* = 0.008). The other strategies were not statistically significant. The model explained 63.2% of the variance (*F* (10,109) = 17.024, *p* < 0.001). The distribution of anxiety and depression severity levels is presented in Figure 2, showing that a substantial proportion of patients experienced moderate‐to‐high emotional distress during CCU hospitalisation. Overall, Personality Type D emerged as a strong predictor of anxiety, depression and HADS total score, with avoidant coping significantly influencing depression and total score. Multicollinearity diagnostics indicated no problematic intercorrelations among predictors, as all VIF values were below the recommended cut‐off of 5.0, supporting the stability of the regression models.

**TABLE 3:** Multiple linear regression analyses predicting anxiety and depression.

Regression model for anxiety	Unstandardised	Standard error	Standardised	*t*	*p*	95% CI	
						Lower	Upper
Intercept	−5787	4854		−1192	0.236	−15 419	3845
Type_D	0.362	0.050	0.700	7171	< 0.001	0.262	0.462
Problem_Focussed_Coping	0.163	0.128	0.125	1278	0.204	−0.090	0.417
Emotion_Focussed_Coping	−0.010	0.095	−0.010	−0.108	0.914	−0.199	0.178
Avoidant_Coping	0.133	0.096	0.113	1390	0.168	−0.057	0.324
*F* (10,109) = 12.971, *p* < 0.001, 56.6%							

Regression model for depression	Unstandardised	Standard error	Standardised	*t*	*p*	95% CI	
						Lower	Upper
Intercept	−2644	3119		−0.848	0.399	−8832	3544
Type_D	0.188	0.035	0.421	5446	< 0.001	0.120	0.257
Problem_Focussed_Coping	−0.218	0.102	−0.193	−2142	0.035	−0.420	−0.016
Emotion_Focussed_Coping	0.103	0.075	0.119	1370	0.174	−0.046	0.253
Avoidant_Coping	0.265	0.076	0.259	3470	< 0.001	0.114	0.417
*F* (10,109) = 18.874, *p* < 0.001, 62.9%							

Regression model for total HADS score	Unstandardised	Standard error	Standardised	*t*	*p*	95% CI	
						Lower	Upper
Intercept	−6729	7467		−0.901	0.370	−21 544	8087
Type_D	0.535	0.078	0.620	6894	< 0.001	0.381	0.688
Problem_Focussed_Coping	−0.052	0.197	−0.024	−0.263	0.793	−0.442	0.339
Emotion_Focussed_Coping	0.088	0.146	0.052	0.600	0.550	−0.203	0.378
Avoidant_Coping	0.399	0.148	0.202	2706	0.008	0.106	0.692
*F* (10,109) = 17.024, *p* < 0.001, 63.2%.							

*Note:* Regression coefficients (β), standard errors (SE) and significance values for predictors of anxiety and depression scores, including Type D personality and coping strategy subscales.

## 6 Discussion

The present study aimed to assess the levels of anxiety and depression among patients hospitalised in the CCU of a regional hospital and to explore their potential correlation with TDP traits and coping strategies. The findings confirm significant associations among these psychological factors and notable correlations with demographic characteristics, underscoring the importance of psychological evaluation in cardiac patient care.

In the present survey, the proportion of men and women was almost equal, reflecting the increased prevalence of CVD in women and demonstrating that the risk of CVD is equalised between the two sexes. This could be attributed not only to the decrease in oestrogen with menopause [22] but also mainly to the modern risk factors, such as unhealthy diet, sedentary lifestyle, stress and especially smoking [23, 24, 25]. In contrast to our finding, the survey of Lee et al. [26] found an overrepresentation of men with acute coronary syndrome (ACS) compared to women.

It was found that more than six out of 10 cardiac patients were tertiary graduates, a finding that leads to the hypothesis that individuals with a higher educational level often work in more stressful professional environments, hold positions of increased responsibility or have a more sedentary lifestyle, conditions that may increase the risk of cardiac diseases. However, this finding is not consistent with those of other studies, where it is reported that individuals with a high educational level have a reduced risk of CVD during their lifetime [27, 28]. It is possible that this inconsistency can be attributed to the small sample of our study and the fact that it was conducted in a single hospital structure.

Another interesting finding that could be discussed is that the average age of patients hospitalised in the unit is 51 years, with 70% of them being employees, an index of the increased incidence of CVDs in younger ages because of the modern and stressful lifestyle [29, 30]. Relevant research reports that the average age of patients admitted to the Unit is approximately 60 years [31]. Similarly, a study in Malaysians reported the development of ACS at a mean age of 58.7 years compared to 63.4 and 68 years in most developed countries [32].

Regarding the history of CHD, it is worth mentioning that almost nine out of 10 participants had no history of CHD before their hospitalisation in the Unit. This fact could also point to gaps in preventative care or screening. Perhaps it is because individuals might not have been regularly screened for heart disease, especially if they did not have obvious signs or symptoms, leading to undiagnosed CHD until a serious event occurs. This finding is significant because it underscores the potential for CHD to develop without prior obvious symptoms or diagnoses, and it may suggest that more proactive healthcare measures, such as regular screening and lifestyle changes, could help reduce the incidence of severe CVD events.

Regarding psychological conditions, it was found that almost half of the patients presented moderate‐to‐high levels of hospital anxiety and depression. This percentage is higher than that of the meta‐analysis, which indicated that three out of 10 patients with CVD present significant levels of clinical anxiety [33]. This finding was partially expected, given that patients in the CCUs often experience high rates of depression and anxiety due to many factors. A diagnosis of ischaemic heart disease, a cardiovascular condition that affects millions of people globally, can cause individuals to enter a state of physical, mental and emotional uncertainty [34]. In addition, physical symptoms associated with heart disease, such as chest pain, dyspnoea and fatigue, can exacerbate psychological distress, leading to increased levels of anxiety and fear of death [35].

In our study, one out of three hospitalised patients had TDP characteristics. Despite that, the percentages of this type of personality reported by other studies are lower [19, 36]; however, it is mentioned that the prevalence of TDP is differentiated, ranging between 20% and 40% across different types of cardiovascular conditions. Type D personality consists of a combination of negative affectivity in stressful situations (NA) and the suppression of its manifestations in social interactions (SI) and is an established risk factor for the development and prognosis of CHD [4, 37, 38]. Studies have demonstrated the role of various behavioural aspects of health in explaining the increased incidence of heart disease and the early development of heart disease having consistently associated TDP with less healthy eating behaviours [39, 40, 41], less regular physical exercise [42, 43], poorer medication adherence [44] and self‐management behaviours [45].

We also found that TDP is a prognostic factor both for anxiety and depression, and it can relate to CVDs. This finding agrees with those of other studies, which found that the respondents with TDP had higher stress and anxiety levels as well as higher negative coping scores than those for positive coping [46, 47]. It is documented that people with TDP have a significant increase in blood pressure, pulse and their amount of cortisol compared to non‐TDPs. Stress is one of the risk factors for CVD, and it also has an association with increased systolic blood pressure. Stress can trigger the release of catecholamines quickly, which will later cause an increase in cardiac output and blood pressure. A comparison study showed that TDP prevalence is usually lower in cardiovascular patients than in the general population [48]; however, some researchers suggest that it is not Type D that is related to worse health, but rather NA [49].

In terms of coping strategies, a positive correlation was also found in this study between avoidance, depression and overall hospital stress scores (HADS). This finding is also in agreement with the wider literature, where it is reported that there is a constant feedback relationship as anxiety and depressive symptoms fuel the avoidance of confrontation with problems, with this avoidance fuelling depression and anxiety again [50]. Avoidance treatment consists of focussing attention away from the problem representing the denial or minimisation of a stressful situation, whereas the person passively decides that nothing can be done to change, dealing with the problem by adopting alternative behaviours (smoking and unhealthy habits), which have negative consequences such as maintaining and aggravating psychological distress [51, 52].

Opposite to the above finding, a negative correlation emerged between depression and problem‐focussed strategies, which are mobilised to manage the stress of patients with heart problems. The negative association of depression with focus on the problem is supported by other research, which reports that depression was positively correlated with avoidance strategies but negatively with problem‐solving strategies, demonstrating that individual coping strategies have their significance concerning depression and can be used to establish an evidence‐based cognitive behavioural approach to depression [53].

The associations between TDP and emotional distress in patients with CKD can be understood through converging psychological and psychophysiological pathways. First, impaired emotion regulation in the context of high NA is consistently associated with higher anxiety/depression. Individuals with Type D display more persistent negative appraisals and difficulty reducing arousal, which exacerbates distress during acute cardiac hospitalisation. In addition, reduced social support and interpersonal inhibition appear to be central. Type D is associated with lower perceived support and greater social withdrawal [4, 54]. Both have been shown to mediate associations between Type D and stress/somatic symptoms and worsen mood outcomes when patients face health threats. In groups of patients after myocardial infarction, social support and coping style partially explained the Type D–distress association, suggesting that inhibition of help‐seeking deprives patients of emotional regulation in high‐stress environments. Finally, maladaptive coping, especially avoidance, likely drives Type D's vulnerability to anxiety/depression. Observational and longitudinal work in cardiology populations suggests that Type D is associated with greater use of avoidance and with depressive tendencies over time. These underlying mechanisms provide a useful framework for interpreting our findings [13, 55]. Designing interventions around screening for Type D, brief emotion regulation skills, supportive mobilisation and coping retraining may therefore be clinically relevant in this context.

Importantly, the expression and clinical impact of these personality and coping patterns may also be influenced by cultural norms surrounding emotional expression and interpersonal restraint. Although the current study is based on a Greek patient sample, the generalisability of the TDP construct and its manifestation across different cultural contexts remains an open question. A large international study comprising 6222 patients across 21 countries established measurement equivalence of the DS14 scale and reported prevalence differences (Southern Europe ~37%, Northern/Western Europe ~24%) [56]. These findings suggest that cultural norms of emotional expression, interpersonal inhibition and coping may shape both the prevalence and clinical relevance of Type D traits. Therefore, caution is warranted when extrapolating our findings beyond the Greek context, and future multi‐national investigations are recommended to elucidate cultural influences on TDP and associated psychological distress.

In patients with CAD, emotional distress is closely linked to poorer self‐care, medication nonadherence and increased hospital readmissions. A clinical perspective, brief psychometric tools such as the DS14 and Brief COPE can be feasibly administered by nurses within the first days of CCU admission. Embedding such assessments into routine nursing workflows requires minimal additional time yet provides high‐value information to guide psychosocial support and multidisciplinary care planning. Early identification of patients with Type D traits could facilitate targeted communication strategies, cognitive behavioural support and early referral to psychosocial services, ultimately improving both emotional and cardiac outcomes.

## 7 Limitations

This study has several limitations. First, its cross‐sectional design limits causal interpretation; however, we addressed this by applying multivariate regression analyses to identify independent predictors and control for confounding factors.

Second, the research was conducted in a single regional hospital with a moderate sample size (*n* = 110), which may restrict generalisability. To mitigate this, we used consecutive sampling and standardised data collection procedures by a single trained nurse to enhance internal validity and reduce selection bias. Additionally, although consecutive sampling was used to reduce systematic selection bias, the recruitment of participants from a single centre limits the generalisability of the findings to broader cardiac populations. Future research should employ multicentre designs with larger and more heterogeneous samples to enhance representativeness and external validity. Third, the use of self‐report questionnaires introduces the potential for social desirability or recall bias. This was minimised by ensuring participant anonymity, providing a supportive environment and using well‐validated Greek versions of the scales with high internal consistency.

## 8 Implications for Practice

This study shows that TDP and avoidant coping are strong predictors of emotional distress in CCU patients. These findings suggest several practical actions for nursing.

First, incorporating brief tools such as the DS14 and Brief COPE into admission assessments can help nurses identify emotionally vulnerable patients early, allowing for timely psychosocial support. This process requires minimal time but yields valuable insight into each patient's emotional needs.

Second, understanding a patient's personality and coping style enables nurses to adapt communication and care strategies. For example, socially inhibited patients may benefit from a calm, non‐judgemental approach that fosters trust and emotional expression, whereas those using avoidant coping may need encouragement to engage actively in recovery.

Finally, applying these insights promotes humanised, holistic cardiac care. By integrating psychological assessment with compassionate nursing practice, nurses can address both emotional and physical dimensions of healing, enhance adherence and improve overall recovery experiences for patients in the CCU.

## 9 Conclusions

Type D personality and avoidant coping emerged as strong predictors of anxiety and depression in CCU patients, underscoring the need for early psychosocial screening in cardiac nursing. Integrating brief tools such as the DS14 and Brief COPE into nursing assessments can help identify emotionally vulnerable patients and guide targeted support. Although limited to one site, this study, the first of its kind in Greece, provides evidence that nurse‐led personality profiling is both feasible and clinically valuable. Future longitudinal research should confirm these findings and evaluate interventions that enhance adaptive coping and emotional recovery in cardiac populations.

## Author Contributions

**Anastasia Lykou:** conceptualisation, methodology, investigation, writing – original draft. **Evangelos C. Fradelos:** validation, writing – review and editing, resources. **Ioanna Dimitriadou:** project administration, writing – review and editing. **Maria Saridi:** data curation, formal analysis, visualisation. **Eustratia Mourtou:** validation, writing – review and editing, resources. **Pavlos Sarafis:** formal analysis, visualisation, methodology. **Dimos Mastrogiannis:** formal analysis, visualisation, methodology. **Ioanna V. Papathanasiou:** software, investigation, data curation. **Aikaterini Toska:** supervision, funding acquisition, writing – review and editing.

## Funding

The authors received no specific funding for this work.

## Ethics Statement

The study was conducted in accordance with the ethical principles outlined in the Declaration of Helsinki. Ethics approval was obtained from the Scientific Committee of the General Hospital of Rhodes (Protocol No. 28056/8‐10‐2023).

## Consent

All participants were fully informed about the purpose and procedures of the study and provided written informed consent prior to participation.

## Conflicts of Interest

The authors declare no conflicts of interest.

## Data Availability

The data that support the findings of this study are available from the corresponding author upon reasonable request.
